# Phenotype-based drug screening: An *in vivo* strategy to classify and identify the chemical compounds modulating zebrafish M-cell regeneration

**DOI:** 10.3389/fmolb.2022.984461

**Published:** 2022-10-24

**Authors:** Ankita Kumari, Xin-An Zeng, Abdul Rahaman, Muhammad Adil Farooq, Yanyan Huang, Mahafooj Alee, Runyu Yao, Murtaza Ali, Ibrahim Khalifa, Omnia Badr

**Affiliations:** ^1^ School of Food Science and Engineering, South China University of Technology, Guangzhou, China; ^2^ Guangdong Key Laboratory of Food Intelligent Manufacturing, Foshan University, Foshan, Guangdong, China; ^3^ Overseas Expertise Introduction Centre for Discipline Innovation of Food Nutrition and Human Health (111 Centre), Guangzhou, China; ^4^ Department of Food Science and Technology, Khwaja Fareed University of Engineering and Information Technology, Rahimyar Khan, Punjab, Pakistan; ^5^ Food Technology Department, Faculty of Agriculture, Benha University, Qalyubia, Egypt; ^6^ Department of Genetics and Genetic Engineering, Faculty of Agriculture, Benha University, Qalyubia, Egypt

**Keywords:** phenotype drug screening (PDS), regeneration, zebrafish, axon, M-cell

## Abstract

Several disease-modulatory FDA-approved drugs are being used in patients with neurodegenerative diseases. However, information on their toxicity-related profiles is very limited. Therefore, measurement of drug toxicity is essential to increase the knowledge of their side effects. This study aimed to identify compounds that can modulate M-cell regeneration by causing neuro-protection and -toxicity. Here, we developed a simple and efficient *in vivo* assay using Tg (hsp: Gal4FF62A; UAS: nfsB-mCherry) transgenic zebrafish larvae. Interestingly, *via* the phenotype-based drug screening approach, we rapidly investigated 1,260 compounds from the United States drug collection and validated these in large numbers, including 14 compounds, that were obstructing this regeneration process. Next, 4 FDA-approved drugs out of 14 compounds were selected as the lead hits for *in silico* analysis to clarify their binding patterns with PTEN and SOCS3 signaling due to their significant potential in the inhibition of axon regeneration. Molecular docking studies indicated good binding affinity of all 4 drugs with the respective signaling molecules. This may point to PTEN and SOCS3 as the signaling molecules responsible for reducing axon regeneration. Moreover, the acute effect of compounds in reducing M-cell regeneration delineated their toxic effect. In conclusion, our *in vivo* along with *in silico* screening strategy will promote the rapid translation of new therapeutics to improve knowledge of the toxicity profile of approved/non-approved drugs efficiently.

## Introduction

Over the past few decades, substantial progress in phenotype-based drug screening (PDS) strategy has led to the discovery of FDA-approved first-class small molecule drugs (SMDs). In the period from 1999 to 2008, 78% of first-class SMDs has discovered through this strategy related to the central nervous system (CNS) (Brodie 2010; Swinney and Anthony 2011). Currently, people are adopting the PDS strategy to alleviate disease conditions that have little/zero options for a cure (Childers et al., 2020). Drug discovery through PDS offers ways to develop principle compounds capable of halting and reversing the progression of the disease etiology (Lee and Bogyo 2013). However, due to incomplete knowledge about the disease, phenotype-based drug screening (PDS) strategies are alluring, specifically because they do not need prior knowledge of either particular drug target or an understanding of the mechanism of action (Moffat et al., 2017). Among all diseases, neurological disorders are the utmost area of dispute for drug discovery and its progress due to the inadequate information on complex functional networks of neurons and short of translational ability (Gribkoff and Kaczmarek 2017). Together with this, it is hard to get a reliable and consistent phenotype correlated with particular disease conditions for PDS.

Although in the case of neurology, quantifying phenotype (such as axonal length, alteration in the expression of fluorescently tagged proteins, and viability of neurons) specifically provides a system to produce a useful outcome for the therapeutic study of neurological disorders (Galluzzi et al., 2008; Kepp et al., 2011; Galluzzi et al., 2018). Importantly, it is extremely advantageous to monitor the deleterious effects of drugs before their approval (Friese et al., 2019). By employing the PDS strategy on SMDs library, small molecules can be accurately separated into different groups based on their various phenotypic responses in biological models. These profiled SMDs can be clustered based on hits involved not only in the modulation of disease pathways but also in deleterious effects such as toxicity, low membrane permeability, and lack of biological activity (Frantz 2005; Swinney and Anthony 2011). To run the unbiased screening of small molecules, it is essential to have a physiological environment of neuronal communication together with coordination of endocrine signaling (i.e., these features belong to the animal model). Evolving new approaches, pooled with modern microscopic technologies are resolving the entire wiring illustration of the nervous system (Pankevich et al., 2014). Therefore, animals that share most of their neuro-morphological and biochemical characteristics with humans may help to identify potential clinical approaches to axon recovery (Howe et al., 2013). In the race of learning to improve the functional recovery of axons, various studies showed the physiological conservation between humans and zebrafish, along with drug metabolic pathways and disease-associated targets which share 82% similarity (MacRae and Peterson 2015). Various compounds have been identified in zebrafish screens and shown to have equivalent effects in rodents and humans (Rennekamp and Peterson 2015). From the viewpoint of the pharma industry, zebrafish is a well-grounded experimental model due to its contribution to PDS. In recent years, it has participated in the establishment of three successful therapeutic applications first, in the validation of drug targets recognized by patients sample; second, in the generation of disease models to understand disease mechanisms; and third, in the conduction of PDS for identification of new therapeutics (Cornet et al., 2018). Additionally, optical visuality and genetic tagging with fluorescent markers permit extraordinary optical access to larval Zebrafish *in vivo* CNS and neuronal networks (MacRae and Peterson 2015).

To analyze the influences of PDS on *in vivo* axonal morphology, M-cell (Mauthner cells) is an outstanding experimental model which is part of CNS; it projects from the hindbrain and covers up to the tail region. M-cell is a pair of large bilateral reticulospinal axons (2 M-cells present in each fish) (Eaton et al., 2001; Korn and Faber 2005). Its large size, finite numbers, and its easy visualization through genetically directed reporter/electroporation with fluorescent dye/fluorescent coding plasmid make it a crucial model for neurological studies (Bhatt et al., 2004; Satou et al., 2009; Feng et al., 2010). Zebrafish’s M-cell has robust axonal regeneration ability; through *in vivo* imaging technique in the intact animal, it is interesting to obtain mechanistic insights into this process (Hecker et al., 2020). However, it is not easy to uncover autonomic/non-autonomous factors influencing the recovery of injured axons *in vivo* as damage to both the nerve and surrounding tissue is related to the injury (Rieger and Sagasti 2011; Tedeschi and Bradke 2017). Since, initially, the aim of this study was to identify promising compounds that could modulate nerve regeneration by the PDS strategy. However, unfortunately, we did not find any compound that enhances regeneration capacity. But interestingly, meanwhile, we identified various drugs that were causing other phenotypic effects along with reducing regeneration. In this way, we classified the drugs based on their effects on M-cell regeneration.

The PTEN negatively regulates the PI3K-AKT-mTOR pathway, a determinant of intrinsic axonal regeneration ability due to their involvement in metabolism, cell growth, survival, proliferation, and motility (Zhang et al., 2018). Likewise, deletion of SOCS3 (suppressor of cytokine signaling 3) which is a negative regulator of JNK signaling and stimulator of the JAK/STAT pathway, promotes nerve regeneration (Sun et al., 2011). Therefore, we hypothesized that the downregulatory effect of drugs could be due to interaction with PTEN and SOCS3, as both are strong inhibitors of CNS axon regeneration. Therefore, we performed the computational docking analysis to predict signaling molecules through which four FDA-approved drugs may exert a negative impact on axon regeneration.

## Materials and methods

### Zebrafish transgenic lines and their care

To investigate the effect of compounds on M-cell regeneration in live zebrafish (*Danio rerio*), M-cell (Mauthner-cell) was labeled genetically by the UAS-GAL4 system that has been proved non-toxic for zebrafish. Tg62A transgenic *Tg(hsp:Gal4FF62A)* (Yamanaka et al., 2013) and *Tg* (*UAS:nfsB-mCherry*) (Chung et al., 2013) were used in this study. Zebrafish were maintained and bred in constant conditions, by following standard guidelines for fish care and maintenance protocols (Westerfield 2000).

### Laser axotomy of zebrafish larvae and screening strategy

At the 3 days post-fertilization (dpf), zebrafish larvae were anesthetized in 0.02% Tricaine (Sigma-Aldrich#MKCJ6340) and mounted in the lateral position with the help of 3% Methyl Cellulose (MC). Prior to the beginning of the experiment, we checked whether three or four larvae in each well of 96 well plates could survive without having any defect in growth. Further, we incubated the larvae in egg water for 48 h. After 48 h of post-observation, three larvae groups had no abnormality issues but in four larvae groups 30% of the larvae were dead, so we selected 3larvae/well for each compound. Importantly, we considered 1% DMSO as the control group. To monitor the length of the regenerating axon, laser axotomy was performed at a fixed position i.e., 5th somite from yolk extension (towards anterior body part) of 3 dpf larvae by utilizing 100% current and 1300 µs pulse. Physically disconnected axotomized M-cell was verified under a fluorescence microscope by seeing the gap between the proximal and distal parts. After the injury, larvae were washed properly in the egg water and transferred to 96 well plates that contained chemical compounds added to egg water. Further, these 96 well plates were incubated at a 28.5°C incubator for up to 48 h.

### Small molecule library and administration

We screened 1,280 compounds available in United States Drug Collection purchased from Microsource Discovery System Inc. (United States) [MicroSource Discovery Systems, Inc.—Home (msdiscovery.com)]. To test their effects on M-cell regeneration, these commercially available compounds were selected to cover a wide range of biological processes. However, in this phenotypic screening, we blindly exposed the larvae to these compounds just after axotomy. Compounds were provided in 100% DMSO solution with a stock concentration of 10 mM and kept at −80°C until use. Before the start of the experiment, the whole protocol was developed and optimized to screen the complete compound library. The working/screening concentrations of compounds were prepared in egg water including 1 µM methylene blue and 0.2 mM N-phenylthiourea (PTU). Although we used 100 µM as an initial screening concentration, few compounds at this concentration were causing death or abnormalities (e.g., curving of the tail, enlargement of yolk sac) in the larvae. Therefore, to reduce the toxicity of these compounds, the stock solution of compounds was further diluted in egg water up to 25 µM and 5 µM, respectively.

### Zebrafish larvae axon regeneration analysis

As the M-cell of Zebrafish larvae has a strong regeneration ability after an injury, our purpose of the screening was to identify the compound that would affect the regeneration process. Consequently, it was essential to define the particular time course of the complete regeneration event of M-cell in our experimental setting. During the observation, we found that distal portions of transected M-cell started fragmenting after 8–9 h of post transection, and complete fragmentation was achieved within 24 h of post transection. Moreover, we decided to observe the screening results at the 48 h of post-treatment by counting the somite numbers. Since this is the time when the complete axon regeneration is not achieved, 25–30% of the distance is yet to be covered. This period is sufficient to separate M-Cell’s own and drug-induced regeneration, as well as it is also beneficial in identifying chronic or over-exposure-inspired poisoning profiles.

For observation of M-cell regeneration at 48 h post-treatment, treated larvae were anesthetized in 0.02% Tricaine (Sigma-Aldrich#MKCJ6340) and mounted in 1% low melting agarose in embryo medium. All analysis and imagining were performed from lateral views of the spinal cord. We observed the length of the regenerating M-cell by counting no. of somites next to the injury site. In this study, regeneration length refers to the maximum regenerated axon length of one or both branches of the M-cell, whereas total regeneration length refers to the sum of the lengths of regenerated axon branches of all larvae in a group.

### Data retrieval, pre-process, and molecular docking

The amino acid sequence of 225 amino acids length of Suppressor of cytokine signaling 3 (SOCS3) protein was retrieved in FASTA format from the UniProt database, with the unique accession number O14543 (https://www.uniprot.org/uniprot/O14543).

Further, protein BLAST was performed to get an identical template for further structure prediction. 2BBU protein template was chosen to have 95.73% identity with the query sequence. The template structure was further implemented to model tertiary structure, comparative modeling approach using the MODELLER 9.21v structure was modeled. For another protein, we have accessed the protein databank to retrieve the crystal structure of (phosphatase and tensin homolog) PTEN by using PDB id-5bzx. Both the proteins were further preprocessed, and energy was minimized using SPDB viewer to reduce structural hindrance and complexity. The modeled protein structure was also validated using the PROCHECK web server which provides a Ramachandran plot. And 3D structure file of four drugs (Medroxyprogesterone Acetate, Mifepristone, Quetiapine, and Warfarin) was obtained from the PubChem database.

The computational approach for molecular docking simulation was done using AutoDock Vina software. The suitable format files of both proteins and all ligands to perform molecular docking were prepared through AutoDock tools in the PDBQT format. Non-polar hydrogen atoms were added while generating PDBQT files of the receptor protein. PyMOL and Discovery studio visualizer tools were used to visualize docking results and interaction analysis among residues or atoms of receptor protein and drug ligand.

### Statistical analysis

All data represented in the graphs are in terms of mean ± S.E.M. Paired comparison test was applied to compare the significant (*p < 0.05, **p < 0.01, ***p ≤ 0.001) and non-significant (n.s.) differences within control and experimental groups.

## Results

### Screening strategy and analysis

**FIGURE 1:**
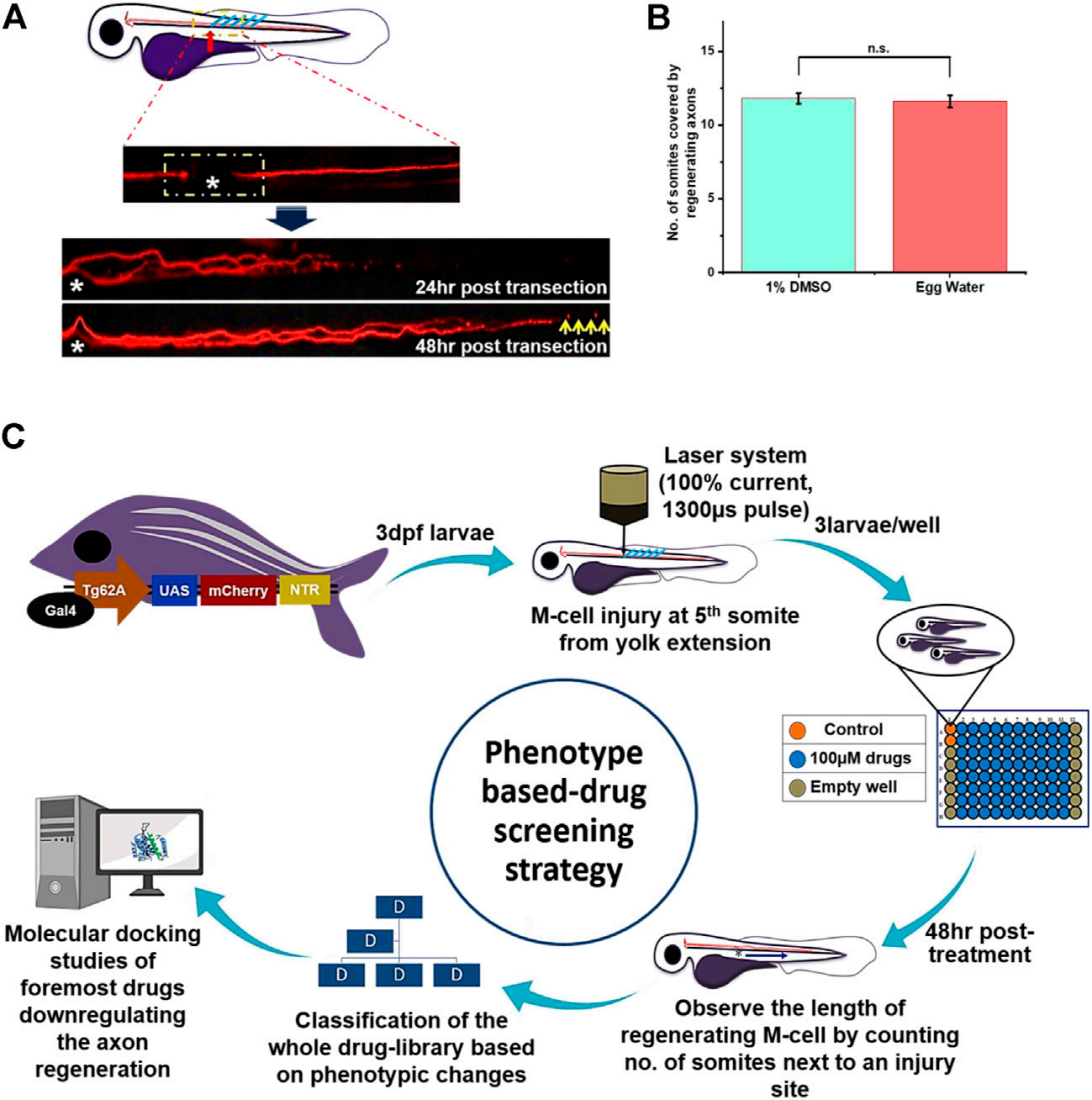
Schematic representation of phenotype-based drug screening strategy: **(A)** Before the start of the experiment, we chose the laser axotomy for consistency of the phenotype. Each time confirmation of the axotomized M-cell was done by seeing the gap between the proximal and distal part of the axon under the fluorescence microscope. The time course of the M-cell regeneration and time-point for result observation was decided by observing the whole regeneration process starting from 8 h to 56 h till the completion of the regeneration process. At 48 hr-post-axotomy, recovery of M-cell regeneration would not be completed and still, it has few distances to recover (as shown by the yellow arrow). So we selected this time point to observe the PDS results. **(B)** Next, we observe that wild-type larvae treated with 1% DMSO and egg water had no significant difference in axon regeneration, and M-cell usually recovered 10–14 somites within 48 hr-post-axotomy. So less than eight somites are assumed to be slow and more than 14 are assumed to be fast regenerating M-cell. **(C)** The plan of action for phenotypic drug screening starts with laser axotomy of M-cell in 3dpf larvae of *Tg (hsp: Gal4FF62A; UAS: nfsB-mCherry).* To fix the position of axotomy in larval zebrafish, each time axotomy was performed at the 5^th^ somite from yolk extension (towards anterior body part). Three larvae were taken in each well; the color of the well shown above presents experimental groups like the orange for the control group, the blue for the compound treated group, and the gray for the empty well. Each time 80 compounds were screened per 96 well plates by leaving rows 1 and 12 empty. Further, based on phenotypic response whole library was classified into three groups and subgroups. Further, four drugs that severely affecting the axon regeneration of M-cell were selected for molecular docking studies with the target proteins to predict the target signaling factors.

We screened 1,280 compounds available in United States drug Collection supplied by Microsource Discovery System Inc. to test their effects on M-cell regeneration. These commercially available compounds were selected to cover a wide range of biological targets. However, in this phenotypic screening, we blindly administered these compounds after axotomy on 3dpf larvae in replicates of three in each well. Compounds were provided in 100% DMSO solution with a stock concentration of 100 mM. Although we chose 100 µM as an initial screening concentration, several compounds at this concentration were causing death or abnormalities (e.g., curving of the tail, enlargement of yolk sac) in the larvae. Therefore, these compounds were further diluted into the egg water up to 25 µM and 5 µM, respectively to minimize toxicity. Prominently, we chose 1% DMSO for the control group because after final dilution every compound was in 1% DMSO. Moreover, it did not cause any toxic effect on axon regeneration Figure 1C. We sought to investigate the effect of compounds on M-cell regeneration in live zebrafish, so M-cell (Mauthner-cell) was labeled genetically by the UAS-GAL4 system that has been proved non-toxic for zebrafish (Distel et al., 2009).

As the M-cell of Zebrafish larvae has a strong regeneration ability after an injury, our purpose of the screening was to identify the compound that would accelerate the regeneration process. Consequently, it was essential to define the particular time course of the complete regeneration event of M-cell in our experimental setting. Wallerian degeneration (Bremer et al., 2019; Hecker et al., 2020) of M-cell i.e. fragmentation and initiation of cell-debris clearance together with regeneration were observed at 8–9 h and 12 h of post-transection, respectively. Most importantly, we also found that cell-debris clearance and regeneration processes coincided. Further, completion of M-cell regeneration was achieved usually after 52–56 h of post-transection. Therefore, we decided to keep 48 h post-treatment, a time point to observe regenerating axons where complete axon regeneration is not expected, as shown in Figure 1A.

### Recovery of axotomized larval M-cell and decision of observation time-course

The robust regenerative capacity of zebrafish M-cells is widely known (Hu et al., 2018; Hecker et al., 2020), nevertheless, it was necessary to define the precise time-course of the M-cell regeneration process after laser axotomy, as our experiment was designed to identify small molecules which could modify the regeneration process. We chose laser axotomy as it is an ideal model for injury and conducts an equivalent injury each time in a specified and controlled way. Therefore, laser axotomy was achieved by utilizing 100% current and 1300 µs pulse near the anterior body part at 5^th^ somite from yolk extension in three dpf Tg62A larvae that injured fewer neighboring cells. Additionally, advanced technology of fluorescent microscopes helped to verify the gap between the proximal and distal axon part of axotomized M-cell Figure 1A. Recovery of the M-cell was assessed *in vivo* from 8 h to 56 hr-post-axotomy using fluorescence microscopes. Further, M-cell began its regeneration following the 12 hr-post-axotomy and got completed following the 52-56 hr-post-axotomy. To measure the length of the regenerating axon, somite numbers covered by regenerating axons were counted. Following 52 h to 56 hr-post-axotomy, most of the larval zebrafish completed their M-cell regeneration. Therefore, we decided to observe the whole screening results at 48 h-post-axotomy, a time point where we can judge comparatively each fast/slow growing M-cell and none of the M-cell would get completed their regeneration. In the control group, we found that most of the larval M-cell recovered 10 to 12 somites next from the injury site following 48 h-post-axotomy Figure 1B. Therefore, we designated eight somites as slow regenerating and 14 onwards as fast regenerating M-cells.

### Phenotype-based drug screening identifies the compounds with down-regulatory properties in axotomized larval zebrafish

To identify the compounds competent to modulate the recovery of axotomized M-cell regeneration process, we developed a phenotypic assay that allowed the screening of the whole compound library including FDA-approved drugs. To perform axotomy, we selected 3 dpf larvae because until 5 dpf they do not require exogenous feed for their survival and development (Wilson 2012). As our observation duration was about 48 h, so it was essential to select this stage. In this PDS strategy, we instinctively administered the small molecules at 100 µM, 25 µM, and 5 µM concentrations respectively, according to the toxicity of the compounds. Additionally, most of the small molecules were causing death or abnormalities in larvae (i.e., curving of the tail, swelling of yolk sac). Therefore, these compounds were diluted further to minimize the toxicity from 25 µM to 5 µM concentrations, respectively. Moreover, for those compounds that were further causing abnormalities in the larvae at 5 µM, we stopped those compounds for furthermore testing. In each experiment, three experimental conditions were generated which included two control groups and one experimental group: 1) axotomized larvae + egg water; 2) axotomized larvae + 1% DMSO in the experimental group; 3) axotomized larvae + small molecules from a library Figure 1C. From an initial pool of 1,260 molecules screened with our plan of action, we performed several repetitions to get hits and at the end, we identified several small molecules that modulated the M-cell regeneration in axotomized larvae.

### Small molecules were classified based on the phenotypic response of M-cell regeneration

**FIGURE 2:**
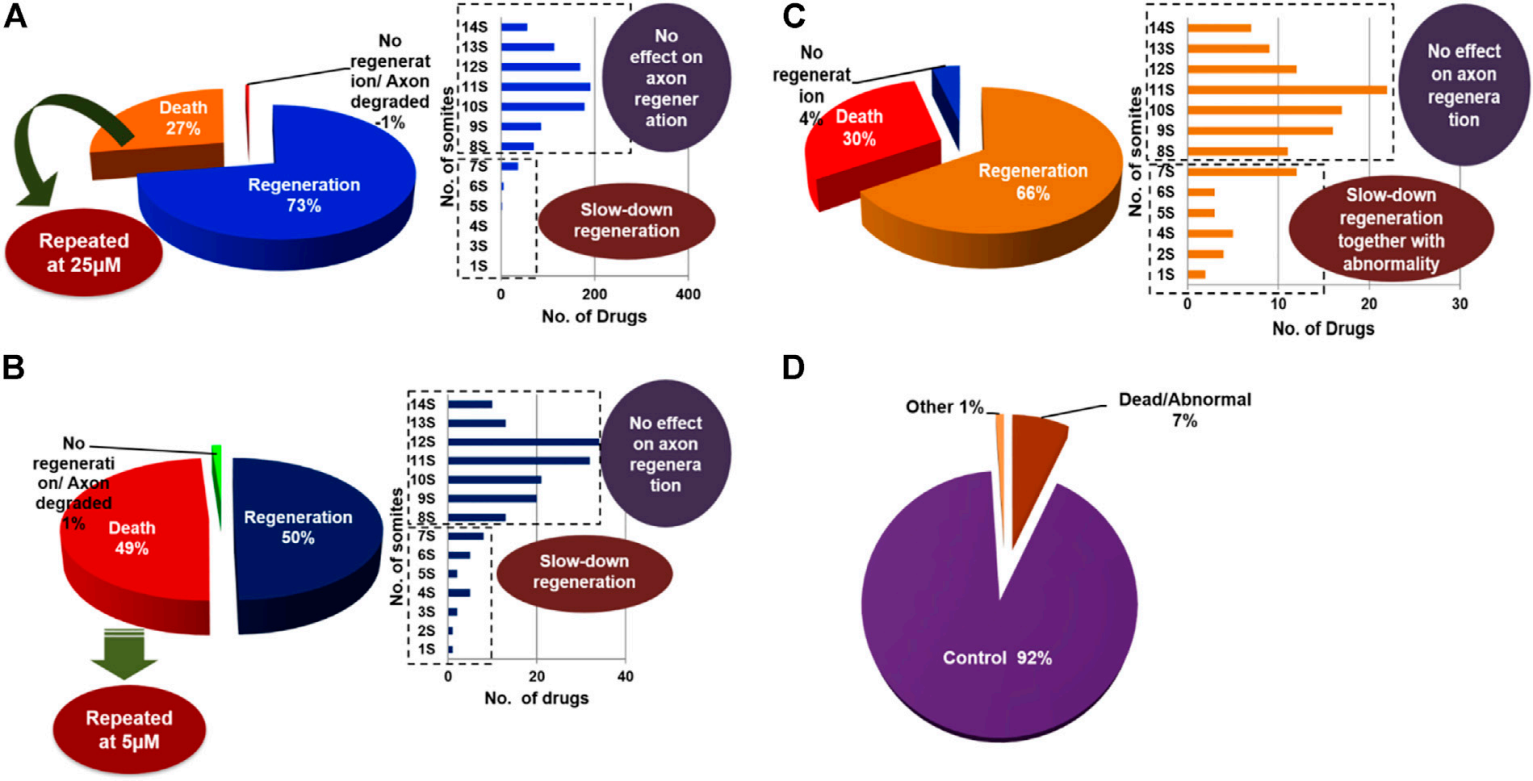
At the initial screening concentration most of the compounds were causing death and were repeated further at a lower concentration. Pie charts are showing the regeneration of M-cell in compound-treated larvae at **(A)** 100 µM, **(B)** 25 µM, and **(C)** 5 µM, respectively. Additionally, bar diagrams are showing regeneration patterns i.e., somite no. of M-cell recovered in treated larvae. **(D)** The pie chart represents the classification of the whole library’s PDS results including all concentrations.

Based on the results obtained from the PDS strategy, we classified the drugs into three categories: 1) regeneration group; 2) No regeneration/degradation of axon group; 3) death-causing group. Further, we again classified the “regeneration group” into two categories: 1) No effect on regeneration group; 2) slow regeneration group. We found that at the 100 μM concentration, 72.0% of the drugs either did not affect regeneration or had a down-regulatory effect on the regeneration process (Figure 2A). Additionally, 27.0% of the drugs were causing larval death, and 1.0% of the drugs had either no regeneration or were causing axon degradation. Interestingly, after diluting this 27.0% of drugs up to 25 µM, we found that 50% of the drugs had a differential effect: 5.9% of drugs had a down-regulatory effect on the regeneration process, whereas 48.8% of the drugs were causing the death of the larvae and 1.2% of drugs had no regeneration or had axon degradation (Figure 2B). Furthermore, to minimize the toxicity of those 48.8% drugs which were causing the death of the larvae at 25 µM concentration, we diluted them up to 5 µM and observed the consequences. Thus, we still got 30% of the drugs that were causing the death of the larvae, so we stopped their further dilution. Apart from this, at 5 µM conc. We found that 66% of drugs had a discrepancy in regeneration, out of that 13.5% had slow regeneration; 4% of drugs either had no regeneration or had axon degradation (Figure 2C). Most of the drugs with down-regulating effects at 25 µM and 5 µM were causing severe instability such as curving of the tail and bulging of the yolk sac. Therefore, we decided to exclude them because down-regulation in the regeneration process could be due to that abnormality. From the beginning, our aim of this study was to identify compounds that could promote regeneration ability; or to identify compounds that are FDA approved but have a down-regulatory effect on M-cell regeneration without causing toxicity. Although we were unlucky enough to cover the first aim, we were successful to achieve the second goal of getting drugs with down-regulatory effects.

### Drugs causing slow regeneration in axotomized larvae

**FIGURE 3:**
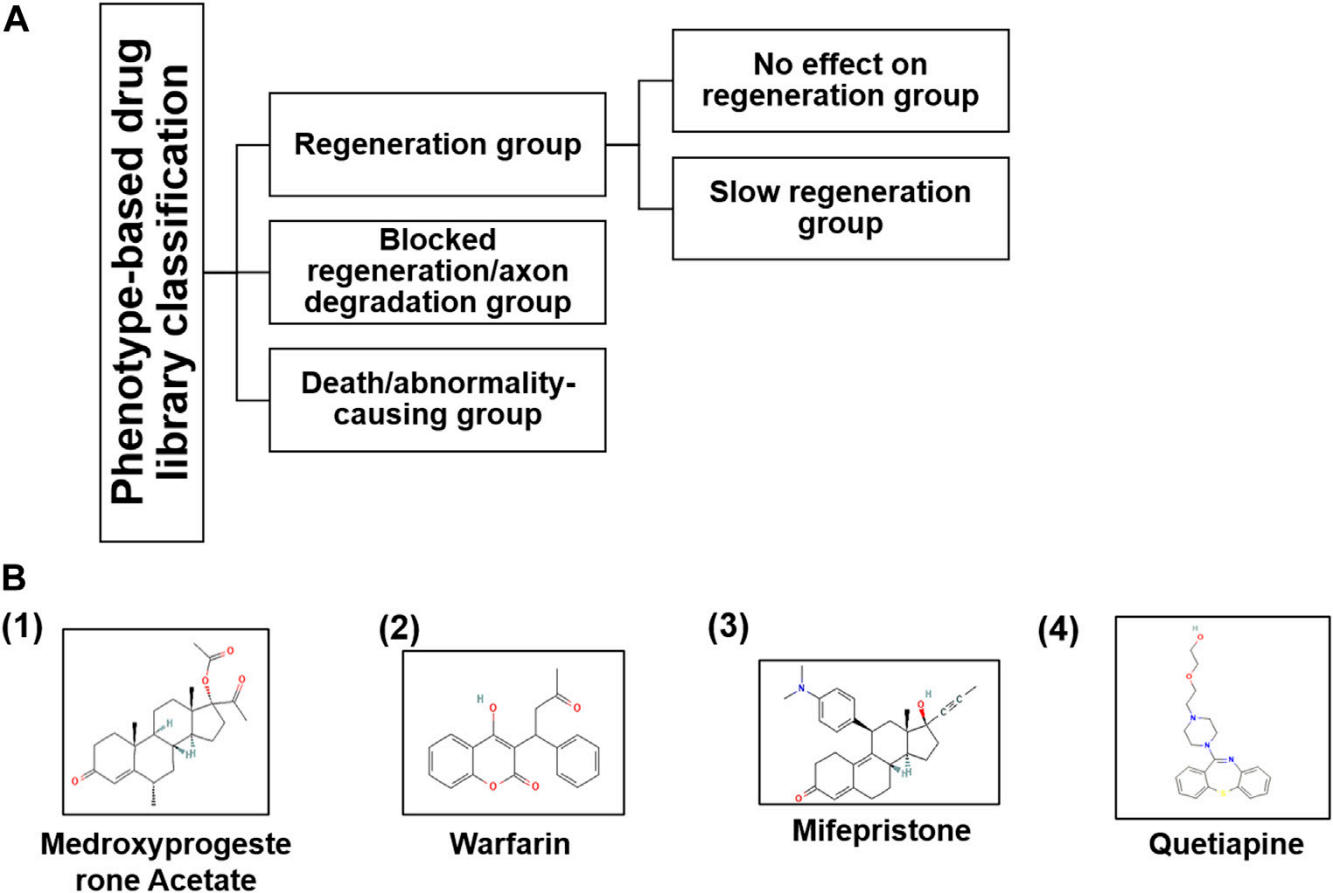
**(A)** Based on the phenotypic responses of M-cells upon drug treatment, we classified the entire library into three categories along with sub-categories and **(B)** their chemical structures taken from PubChem (nih.gov) database.

**FIGURE 4:**
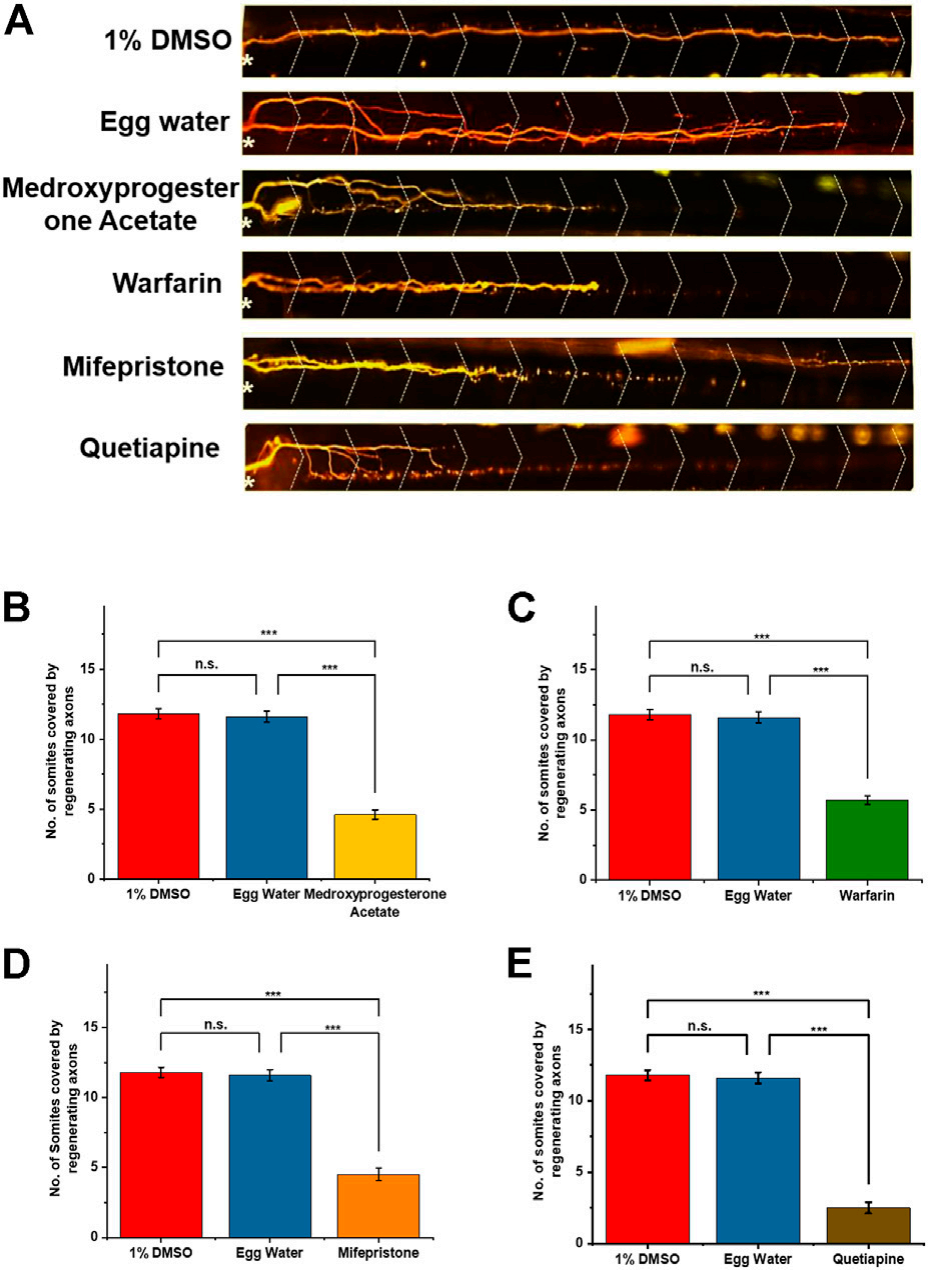
**(A)** Notably, after 48 h of post-axotomy, regenerated M-cells with somite numbers are shown in the figure. The white asterisk (*) is representing the injury point and the dotted greater than (>) is showing the somite structure. **(B–E)** Graphical representation of regenerated axons at 48 h. After treatment: 1% DMSO and egg water as the control group and the effects of different drug treatments are shown *via* bar diagram. Statistical analysis using paired comparison tests (ns, *p < 0.05, **p < 0.01, and ***p < 0.001 between groups and error bars show SD).

Collectively, we found 14 compounds including FDA-approved drugs that had down-regulatory effects on M-cell regeneration, so we selected them for the further screening process. All these drugs had regeneration varied from one somite to six somites and were covering a wide range of biological pathways (Table 2). Out of that, we selected four FDA-approved drugs having a downregulatory effect on M-cell regeneration upon treatment (Table 1). As upon treatment, these four drugs were restricting M-cell regeneration to 3–6 somites compared to the control groups, therefore we selected them for docking studies with well-known inhibitory proteins (PTEN, SOCS3) of CNS axon regeneration. Medicinal goal of drugs and their chemical structures were taken from PubChem (nih.gov) (Figures 3A,B) and their respective effects and statistical analysis have shown in Figures 4A–E.

**TABLE 1:** Representing four drugs and their medicinal use that severely affected larval axon regeneration.

S.No.	Name	Properties
1	Medroxyprogesterone Acetate	A synthetic oral contraceptive, an adjuvant, an inhibitor, an antioxidant, and an antineoplastic agent
2	Warfarin	An oral anticoagulant with anti-vitamin K activity
3	Mifepristone	A potent synthetic steroidal antiprogesterone to induce medical abortion
4	Quetiapine	Antipsychotics for the treatment of schizophrenia and bipolar disorder

**TABLE 2:** List of 14 compounds modulating the regeneration of M-cell in Tg62A larvae: The table includes the compound position, chemical formula, molecular weight, and biological activity along with their effective concentration that down-regulate the M-cell regeneration (Gray shading indicates compounds at 100 µM and yellow one at 25 µM).

S.No.	Chemical name	Well no.	Plate no.	Chemical formula	Mol. Wt	Known biological activity
1	FAMPRIDINE	120,525–09	A04	C5H6N2	94.11697	K channel blocker; multiple sclerosis therapy
2	TERFENADINE	120,525–10	F02	C32H41NO2	471.6891	H1 antihistamine, nonsedating
3	QUETIAPINE	120,525–11	D08	C21H25N3O2S	383.5163	antipsychotic: 5HT antagonist, dopamine antagonist, H1-antihistamine, alpha-adrenergic blocker
4	TICARCILLIN DISODIUM	120,525–12	F08	C15H14N2Na2O6S2	428.3962	antibacterial
5	MIFEPRISTONE	120,525–14	E04	C29H35NO2	429.6078	progesterone antagonist, abortion inducer
6	SULFANITRAN	120,525–15	B05	C14H13N3O5S	335.3408	antibacterial, coccidiostat
7	DEHYDROACETIC ACID	120,525–16	H03	C8H8O4	168.1506	antifungal, antibacterial
8	MERBROMIN	120,525–16	G04	C20H8Br2HgNa2O6	750.6608	antibacterial
9	WARFARIN	120,525–16	D05	C19H16O4	308.337	anticoagulant, rodenticide
10	PERMETHRIN	120,525–16	G07	C21H20Cl2O3	391.2978	ectoparasiticide, CNS stimuant, mutagen
11	DICLORALUREA	120,525–16	H11	C5H6Cl6N2O3	354.8332	antibacterial
12	ACEDAPSONE	120,525–03	F04	C16H16N2O4S	332.3809	antimalarial, leprostatic
13	MEDROXYPROGESTERONE ACETATE	120,525–06	G08	C24H34O4	386.5362	contraceptive
14	NITROMIDE	120,525–06	H08	C7H5N3O5	211.135	antibacterial, coccidiostat

### Structure prediction and molecular docking analysis

**FIGURE 5:**
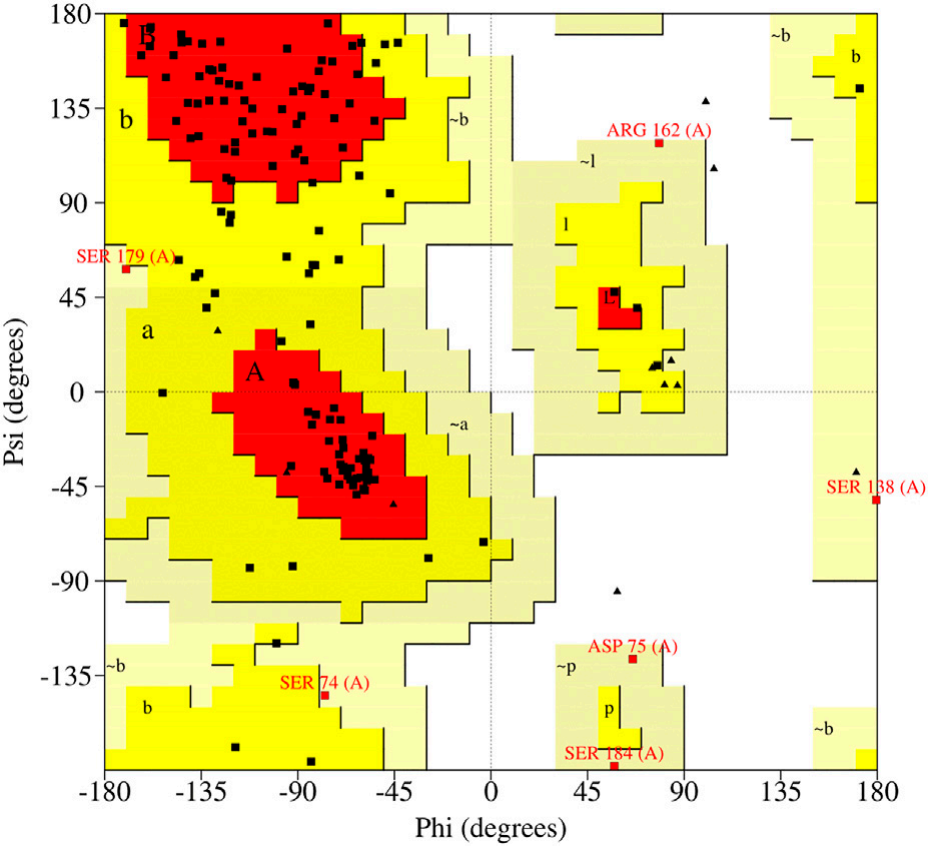
Ramachandran plot shows the statistical distribution of the combinations of the backbone dihedral angles ϕ and ψ.

A preliminary understanding of the interaction between the proteins and four drugs was done *in silico* using various computational approaches such as structure prediction, structure validation, and molecular docking analysis. The 225 amino acid length Suppressor of cytokine signaling 3 (SOCS3) protein was obtained from the UniProt database having a unique accession number- O14543. The three-dimensional structure of this protein is still unknown and therefore a three-dimensional structure of the SOCS3 protein was predicted using the MODELLER tool 9.21v (Webb and Sali 2021) by employing a highly similar template (PDB id- 2BBU) having 95.73% identity that came into BLAST search. The quality of the predicted structure was evaluated using a web-based server, PROCHECK (Laskowski et al., 1993). Ramachandran plot obtained using PROCHECK shown in (Figure 5) represented the distributions of residues and showed the overall good quality of the structure. The plot analysis revealed that a total of 91 (74%) amino acid residues were present in the favored region, 22 (8.3%) in the allowed region, and the rest of 26 (21.1%) and 6 (4.9%) in the generously allowed region. The quantitative values and parameters demonstrated that modeled structure was of fair quality. Moreover, the crystal structure of PTEN protein was obtained by using PDB id-5bzx from the database. The energy was minimized for both proteins to get their actual native form using the SPDB viewer. And small molecules named Medroxyprogesterone Acetate, Mifepristone, Quetiapine, and Warfarin were downloaded from the chemical database PubChem in SDF format and pre-processed using PyRX for the docking process.

**FIGURE 6:**
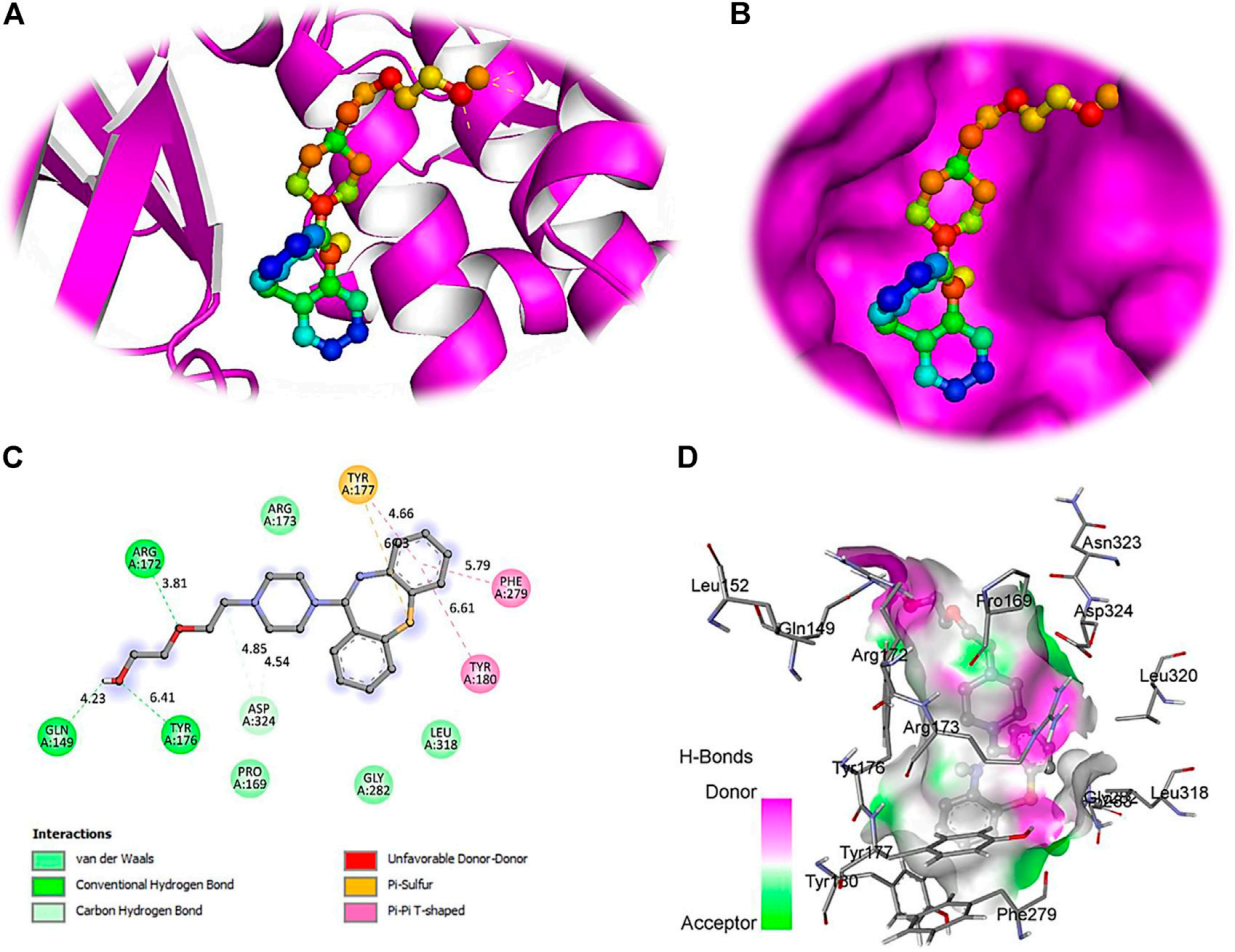
The crystal structure of PTEN in complex with Quetiapine. **(A)** A cartoon representation of PTEN complex close view. **(B)** Surface representation of PTEN in complex with Quetiapine is represented in sticks-boll form. **(C)** A close view of substrate binding pocket representing the key amino acid residues forming interactions with inhibitor molecule. **(D)** Surface representation of conserved substrate-binding pocket of PTEN.

**FIGURE 7:**
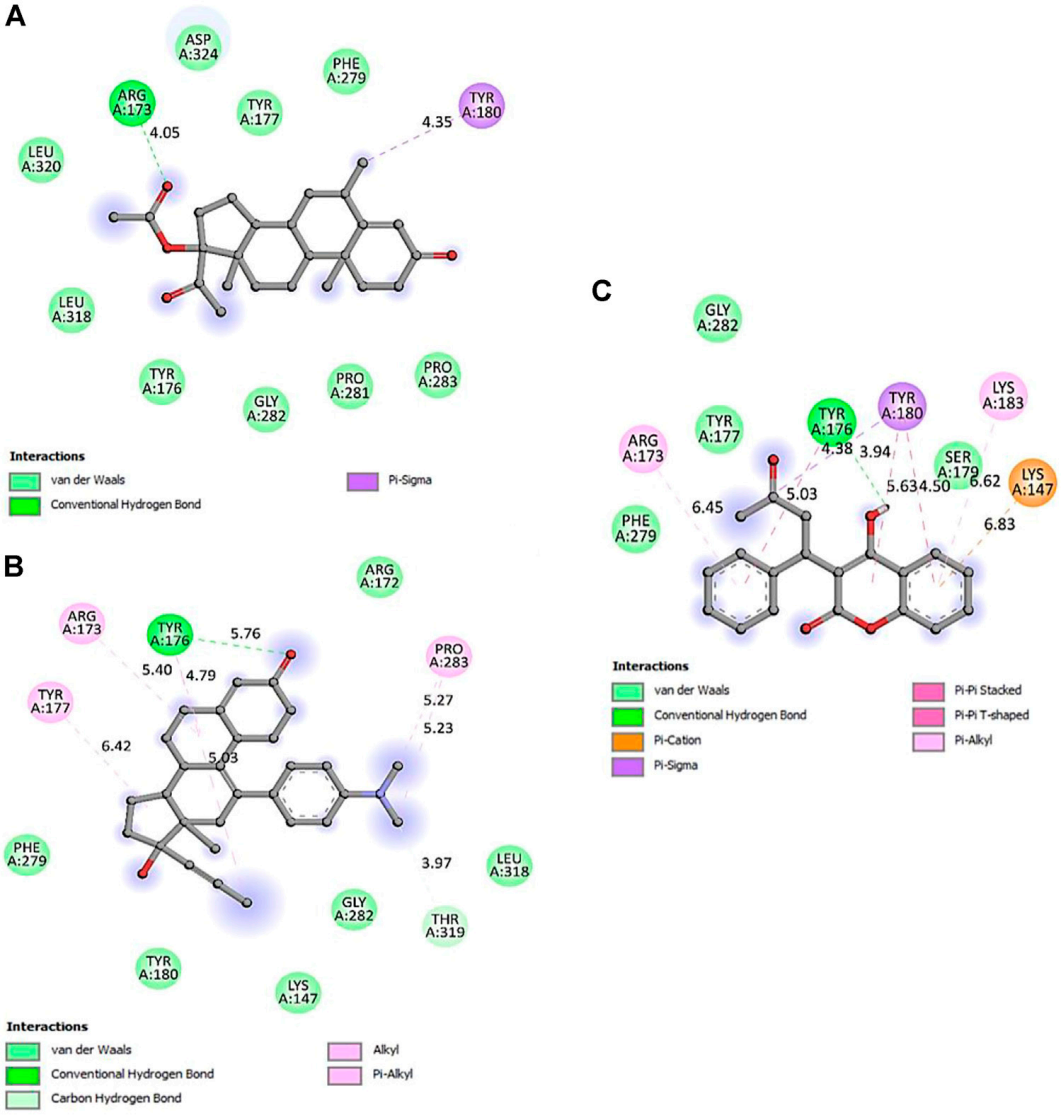
2D representation of PTEN protein-interacting residues in complexed with **(A)**-5bzx-Medroxyprogesterone Acetate, **(B)**-Mifepristone, and **(C)**- Warfarin.

**FIGURE 8:**
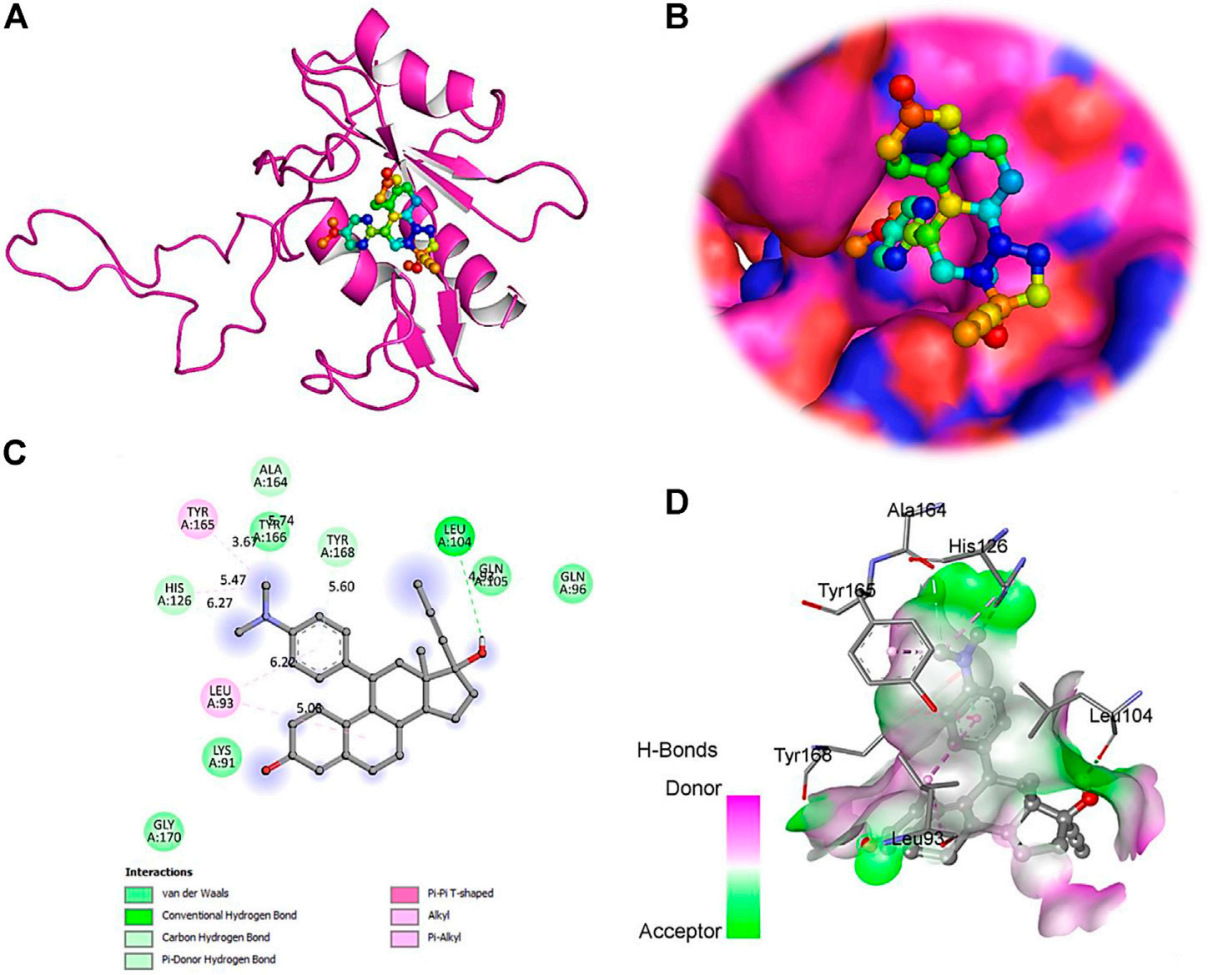
The crystal structure of SOCS3 in complex with Mifepristone. **(A)**- A cartoon representation of PTEN complex close view. **(B)**- Surface representation of PTEN in complex with Mifepristone is represented in sticks-boll form. **(C)**- A close view of substrate binding pocket representing the key amino acid residues forming interactions with inhibitor molecule. **(D)**- Surface representation of conserved substrate-binding pocket of SOCS3.

**FIGURE 9:**
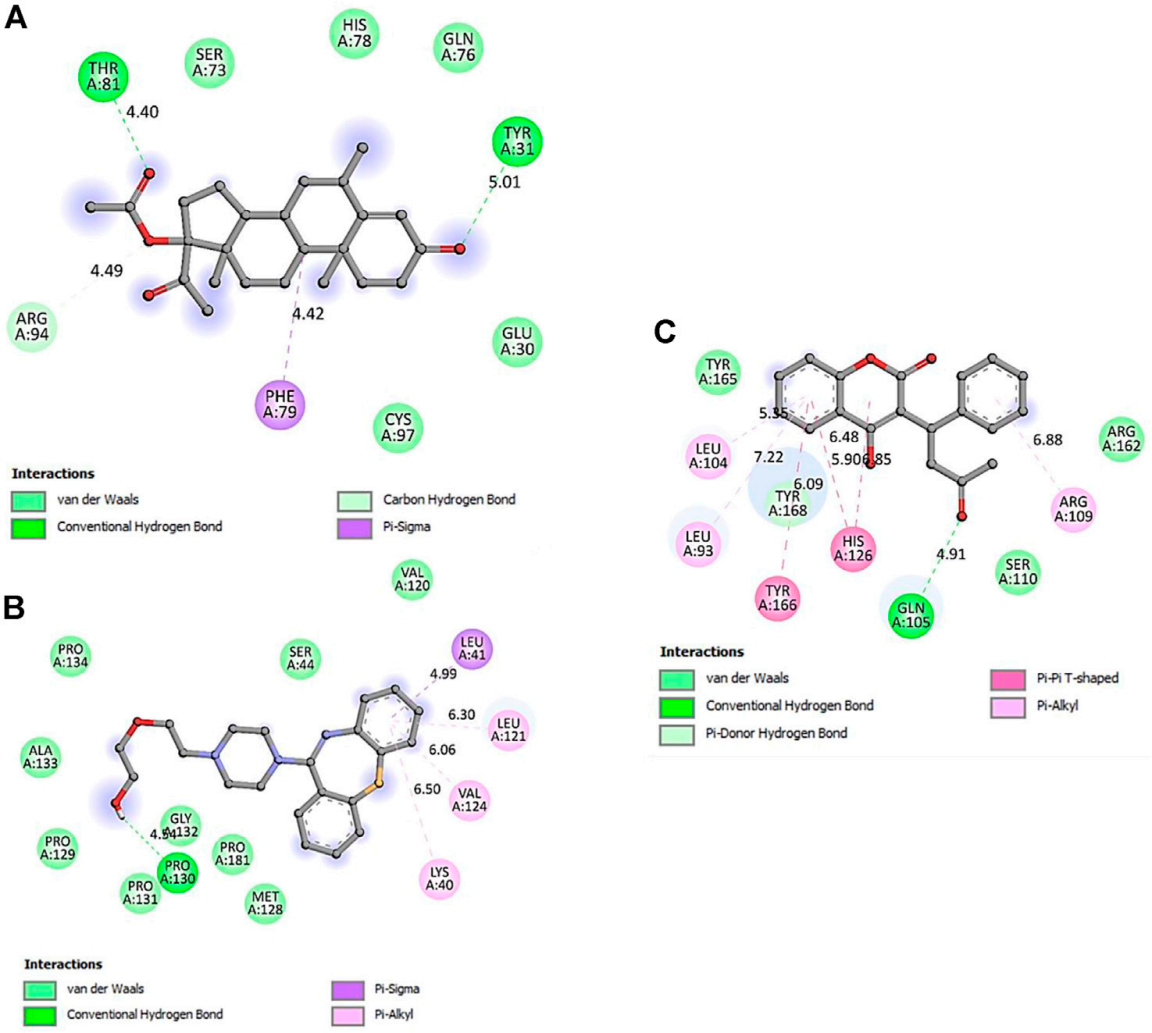
2D representation of SOCS3 protein-interacting residues in complexed with. **(A)**- Medroxyprogesterone Acetate, **(B)**- Quetiapine, and **(C)**- Warfarin.

The computational approach molecular docking simulation was employed using AutoDock Vina software (Trott and Olson 2010). The molecular docking analysis gave deep insight into the interaction between receptor and ligand, the binding energy of receptor-ligand, and the intermolecular distance of the binding residues. We have found that both PTEN and SOCS3 protein binds strongly to all the drugs with the range −6.1–8.2 kcal mol^−1^ binding affinity (Tables 3, 4). Further interaction analysis of all small molecules (Figures 6–9) with both proteins revealed drug Mifepristone formed strong interaction with SOCS3 (Figure 8C) while Quetiapine drug strongly bound with PTEN protein and formed some crucial interactions (Figure 6C). All four drugs shared the same binding cavity of PTEN protein.

**TABLE 3:** Table representing binding affinity and inhibition constant for the interaction of four drugs with SOCS3.

S. No.	Target protein	Compound	Affinity (kcal/Mol)
1	SOCS3	Medroxyprogesterone Acetate	−7.7
2		Mifepristone	−7.6
3		Warfarin	−6.6
4		Quetiapine	−6.1

**TABLE 4:** Table representing binding affinity and inhibition constant for the interaction of four drugs with PTEN.

S. No.	Target protein	Compound	Affinity (kcal/Mol)
1	PTEN	Mifepristone	−8.2
2		Quetiapine	−7.8
3		Medroxyprogesterone Acetate	−7.7
4		Warfarin	−7.4

## Discussion

Phenotype-based drug screening strategy is predominantly important to investigate the drugs modulating a particular disease phenotype even in the absence of specific knowledge of the mechanism of action/validated target compounds (Wagner, 2016; Moffat et al., 2017). Zebrafish larvae are an advantageous animal model that provides important insights into the biological activity of novel chemical compounds through small molecule screenings. Together, its rapid translational ability and expanding gene-editing technology assures to speed up the drug repurposing, and clinical trials (Rennekamp and Peterson 2015). Supporting the aforementioned concepts, we developed a phenotype-based drug screening strategy to investigate drugs from United States drug library collection containing FDA-approved drugs too that can modulate the M-cell regeneration. Because the zebrafish M-cell has the efficient ability to regenerate, so we used 3dpf larvae to perform the axotomy.

Additionally, we chose a laser beam to do injury (100% current and 1300 µs pulse) as laser-induced injury conducts an equivalent injury each time in a specified and controlled way. And in this way, we had the only variable to observe and that was the treatment of different small molecules. Several studies have been performed which elucidate the M-cell regeneration processes (Xu et al., 2017; Hu et al., 2018; Bremer et al., 2019; Hecker et al., 2020). All these studies have supported the dependency of time-course upon the type of injury together with the stage of the larvae and ultimately upon the operator. Thus, it was essential to illustrate the time course of the whole M-cell regeneration process. Our study revealed that the regeneration of M-cell usually recovered within 52-56 hr-post-injury. Further, within the first 8 h–10 h, M-cell started its fragmentation, and following up to 12 h its regeneration initiated. Importantly, it was most needed to choose the time-point at which regeneration of axotomized M-cells was not expected. Further, 48 h is a time of observation was selected to check whether M-cell regeneration was fast or slow.

After deciding the time-point of M-cell regeneration dynamics in our experimental model, we proceeded to execute the phenotypic drug screening where chemical compounds were administered at 3dpf axotomized larvae, and observation of regenerating M-cell was carried out at 48 h-post-injury. We are confident enough about the quality of our zebrafish larvae M-cell experimental model and the planned screening strategy. As the zebrafish larvae, M-cell has robust axonal regeneration ability, so any compound with a down-regulatory effect must have initiated the inhibitory signaling pathway which might be correlated to the human non-regenerating CNS environment. By using the advancement of *in vivo* imaging at the single axon level, we first examined the regeneration pattern of laser-induced axotomized M-cell with 3larvae/well in 96 well plates. Alongside, after each axotomy, we observed 9–12 somites in each group. Consequently, each time we found a consistent phenotype that could be used as an experimental model for PDS.

We showed that 14 drugs had down-regulatory effects on larval M-cell regeneration belonging to different pharmacological classes of molecules. These drugs were down-regulating the M-cell regeneration up to 6 somites from 12 somites (as in the control group). We used the UAS-GAL4 system to label the M-cell; *Tg (hsp: Gal4FF62A; UAS: nfsB-mCherry)* has allowed us to visualize the bilateral reticulospinal neuron. In our phenotypic drug screening, we chose only one concentration for the compounds that were not causing death or abnormalities in the larvae. Further, the death-causing compounds were screened at lower concentrations to minimize toxicity. Thus, those drugs that did not affect M-cell regeneration at a particular concentration would be effective at lower or higher concentrations, respectively. To overcome this problem, one could perform a parallel screen at a lower or higher concentration or optimize another protocol with a short incubation time. Though it is hard to handle a large number of replicates with different drug concentrations and it would significantly influence the cost and time to complete the screening process; consequently, it would lessen the number of hits identified. Here, the compounds modulating the M-cell regeneration at 100 µM were assumed to be active at this concentration, supporting our choice of 100 µM as the primary screening concentration.

Indeed, phenotypic drug screening is advantageous to classify the drugs based on the phenotypic responses (Frantz 2005; Swinney and Anthony 2011). Supporting the above-mentioned concept, we classified the whole library into four groups: First, drugs that did not have any effect on M-cell regeneration; second, drugs that were modulating the M-cell regeneration; third, drugs that were causing degeneration/no regeneration in the M-cell (data has not shown here); fourth, drugs that were causing the death of the larvae (Figure 3A).

Docking studies with PTEN and SOCS3 suggested that Mifepristone builds strong interactions with SOCS3, whereas, quetiapine and the remaining three drugs share the same binding cavity of the PTEN protein. Moreover, molecular docking studies with respective inhibitory proteins explained a theoretical perception for probable molecular interactions of axon regeneration downregulating drugs and target proteins. The strong binding affinity of drugs with proteins may suggest them as a major contributor to downregulating the M-cell regeneration.

Taken all together, in this study we highlighted the prognostic value of the zebrafish larvae as a model for phenotypic drug screening of CNS axon regeneration. Further, we present a well-programmed, simple, and fast phenotypic screening method with the consistent phenotype of M-cell regeneration. Based on the consequences of phenotypic screening we classified the whole drug library into different classes. These results indicated that a wide range of drugs did not have any influence on the M-cell regeneration though a few compounds caused down-regulation in M-cell regeneration. In conclusion, these down-regulatory compounds can create an inhibitory environment by interacting with cellular macromolecules. In summary, we have successfully identified the principles compounds that could influence M-cell regeneration. Furthermore, the study is going on to find out the specific target of those compounds in the cell and their mechanisms.

## Conclusion

During this study, we found that M-cells have a strong regeneration ability on laser axotomy and this unique feature allowed us to analyze the effects of small molecular drugs on CNS nerve regeneration after nerve injury. We used the Tg62A transgenic line of larval zebrafish that stably labels M-cells. Consequently, our findings revealed a down regulatory effect of intensive drugs on M-cell regeneration. Of the 14 drugs known to impair M-cell regeneration, we chose only four because, despite being approved by the FDA, at higher doses, these drugs were reducing axon regeneration capacity, which recommends caution when consuming them. Thus, the present study successfully elucidates the safety/toxicity profile of 1,280 compounds. Docking studies with two widely known inhibitory molecules of axon regeneration advocate that both PTEN and SOCS3 proteins have a strong binding affinity with all four drugs, which persuades these two proteins to reduce axon regeneration. Thus, the present study suggests that PDS analysis with molecular docking studies shows tremendous promise and can significantly improve the usefulness of the PDS strategy to identify drug-induced toxicity assays.

## Data Availability

The raw data supporting the conclusions of this article will be made available by the authors, without undue reservation.
