# Corrigendum: Phenotype-based drug screening: An *in vivo* strategy to classify and identify the chemical compounds modulating zebrafish M-cell regeneration

**DOI:** 10.3389/fmolb.2022.1081922

**Published:** 2023-01-16

**Authors:** Ankita Kumari, Xin-An Zeng, Abdul Rahaman, Muhammad Adil Farooq, Yanyan Huang, Mahafooj Alee, Runyu Yao, Murtaza Ali, Ibrahim Khalifa, Omnia Badr

**Affiliations:** ^1^ School of Food Science and Engineering, South China University of Technology, Guangzhou, Guangdong, China; ^2^ Guangdong Key Laboratory of Food Intelligent Manufacturing, Foshan University, Foshan, Guangdong, China; ^3^ Overseas Expertise Introduction Centre for Discipline Innovation of Food Nutrition and Human Health (111 Centre), Guangzhou, China; ^4^ Department of Food Science and Technology, Khwaja Fareed University of Engineering and Information Technology, Punjab, Pakistan; ^5^ Food Technology Department, Faculty of Agriculture, Benha University, Qalyubia, Egypt; ^6^ Department of Genetics and Genetic Engineering, Faculty of Agriculture, Benha University, Qalyubia, Egypt

**Keywords:** phenotype drug screening (PDS), regeneration, zebrafish, axon, M-cell

In the published article, there was an error in the legend for **Tables 3, 4** as published. The corrected table legends appear below.

TABLE 3 Table representing binding affinity and inhibition constant for the interaction of four drugs with SOCS3.

TABLE 4 Table representing binding affinity and inhibition constant for the interaction of four drugs with PTEN.

In the published article, there was an error in **Table 1 and Figure 3** as published. Table 1 and its caption were duplicated in Figure 3. The corrected Figure 3 and its caption appear below.

**FIGURE 3:**
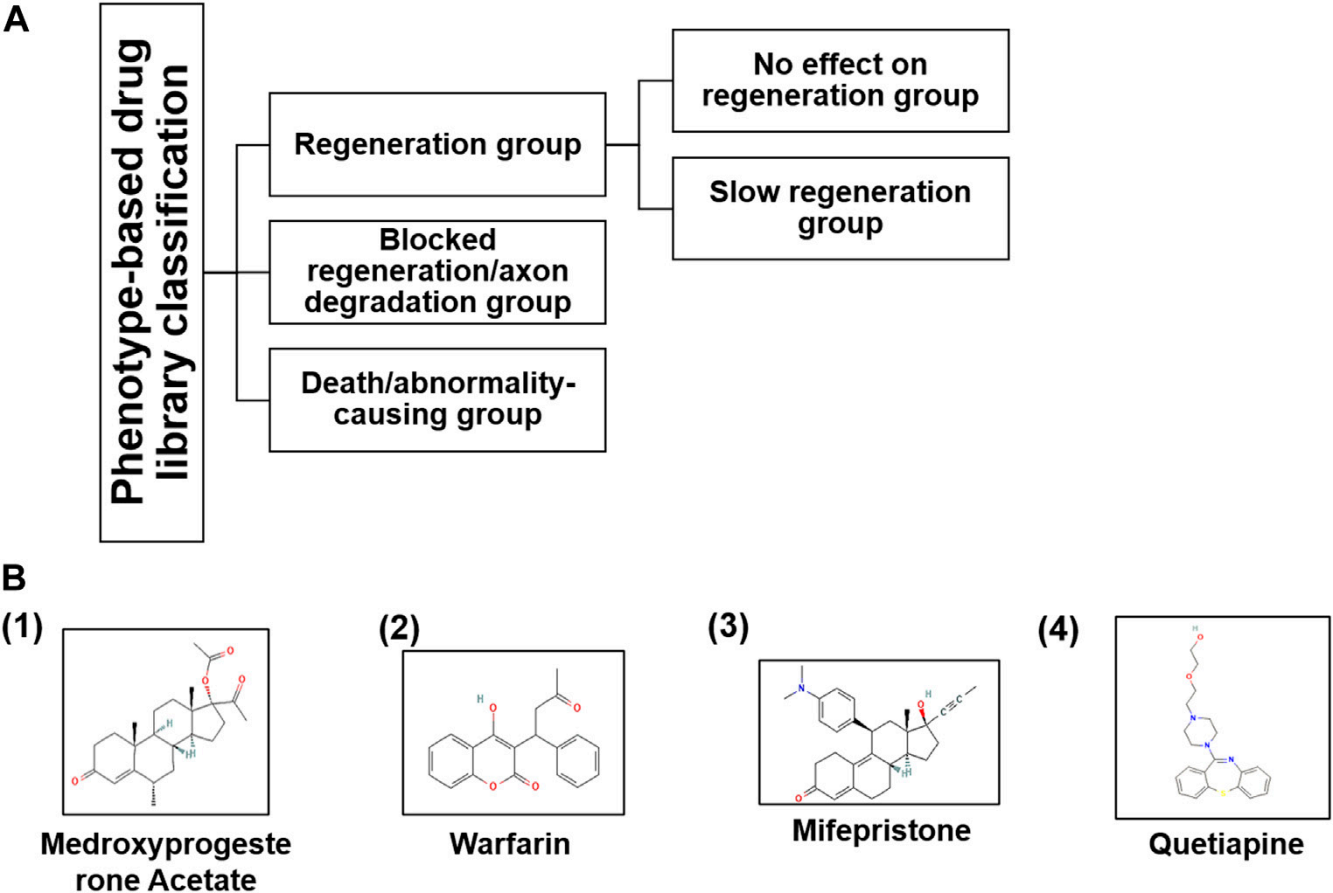
**(A)** Based on the phenotypic responses of M-cells upon drug treatment, we classified the entire library into three categories along with sub-categories and **(B)** their chemical structures taken from PubChem (nih.gov) database.

The authors apologize for this error and state that this does not change the scientific conclusions of the article in any way. The original article has been updated.

